# Classification of Promoter Sequences from Human Genome

**DOI:** 10.3390/ijms241612561

**Published:** 2023-08-08

**Authors:** Konstantin Zaytsev, Alexey Fedorov, Eugene Korotkov

**Affiliations:** 1 Bach Institute of Biochemistry, Federal Research Center of Biotechnology of the Russian Academy of Sciences, 119071 Moscow, Russia; 2 Institute of Bioengineering, Federal Research Center of Biotechnology of the Russian Academy of Sciences, 119071 Moscow, Russia

**Keywords:** human genome, promoter, genetic algorithm, multiple alignment

## Abstract

We have developed a new method for promoter sequence classification based on a genetic algorithm and the MAHDS sequence alignment method. We have created four classes of human promoters, combining 17,310 sequences out of the 29,598 present in the EPD database. We searched the human genome for potential promoter sequences (PPSs) using dynamic programming and position weight matrices representing each of the promoter sequence classes. A total of 3,065,317 potential promoter sequences were found. Only 1,241,206 of them were located in unannotated parts of the human genome. Every other PPS found intersected with either true promoters, transposable elements, or interspersed repeats. We found a strong intersection between PPSs and Alu elements as well as transcript start sites. The number of false positive PPSs is estimated to be 3 × 10^−8^ per nucleotide, which is several orders of magnitude lower than for any other promoter prediction method. The developed method can be used to search for PPSs in various eukaryotic genomes.

## 1. Introduction

Promoter regions are important for RNA transcription [1]. Such regions are located upstream of DNA coding regions and serve to bind RNA polymerase and several other proteins involved in transcriptional regulation [2]. Eukaryotic promoters can contain highly conserved motifs such as the initiator (Inr), TATA box, DPE, and the bridge, a bipartite core promoter element [3]. These motifs are located in the region −499 to +100, with the +1 position being the transcript start site (TSS). For the positioning of the TSS, sequencing technologies are used that allow for rather precise positioning [4]. The use of OligoCap, CAGE, and deepCAGE makes it possible to find TSSs in in vivo experiments [5,6]. However, the use of experimental methods is still expensive. Therefore, it is easier to use bioinformatics methods for promoter search in sequenced genomes instead [7]. Mathematical methods for promoter search have developed significantly in the last 20 years. Promoter sequence search can greatly improve the accuracy of predicting genes and DNA coding regions [8], as well as accurately locate TSS positions. TSS coordinates are particularly important for genetic engineering and personal medicine because gene expression is regulated by promoter activation and inhibition by various proteins [9,10].

Promoter sequence recognition can be performed using neural networks trained on the existing set of promoters [11], such as the EPD database [12,13], because all promoters in it have been experimentally verified. If the structure and relative positions of individual promoter motifs are known, mathematical tools such as Context Free Grammar can also be used for promoter search [14]. However, promoter sequences are very diverse, which makes their prediction difficult. To overcome this diversity, prokaryotic promoters were divided into several classes based on the sigma factor. Promoter classification allowed us to reduce the number or false positives for promoter prediction by adjusting the search methods for each class [15,16].

However, all of these methods have a common flaw. They produce a large number of false positives that can interfere with true promoter identification. Even the best of them will make a false positive prediction every few tens of thousands of nucleotides [17,18,19,20,21,22,23]. This would result in more than 3 × 10^5^ false positive predictions for the human genome. There are approximately 3 × 10^4^ known promoters in the human genome [12], so such a number of false positives would be an order of magnitude higher than for true promoter sequences. Due to the difficulty in identifying true promoters from the false positives, there is a need for other promoter prediction methods with higher sensitivity for the analysis of complete eukaryotic genomes.

To solve the promoter diversity problem, we used the MAHDS method [24] for the multiple alignment of promoter sequences, which is based on the genetic algorithm and dynamic programming [24]. Its main advantage is the ability to compute a statistically significant multiple alignment for nucleotide sequences that have accumulated a large number of nucleotide substitutions relative to each other. Other methods such as ClustalW [25], Clustal Omega [26], MAFFT [27], T-Coffee [28], and Muscle [29] can only produce statistically significant multiple alignments for sequences with *x* < 2.4, where *x* is the average number of substitutions per nucleotide [30] among the aligned sequences. This is due to the fact that these methods generate multiple alignments using pairwise alignments in one way or another. When *x* > 2.4, pairwise alignments become statistically insignificant and there is no way to generate statistically significant multiple alignments from them. However, statistically significant multiple alignments can exist for *x* > 2.4 and can be generated for a number of sequences greater than 2. This topic has been discussed in detail in [24]. 

Unlike other methods, MAHDS is able to generate multiple alignments for sequences with *x* < 4.4 [24,30]. The reason is that MAHDS uses multiple alignment patterns instead of generating multiple alignments from pairwise alignments. For promoter sequences, *x* ≈ 3.6 [24]; so, statistically significant multiple alignments cannot be obtained with any method other than MAHDS. 

The MAHDS method has previously been used for promoter prediction in the *Arabidopsis thaliana* and *Oryza sativa* genomes [24,31]. The number of false positives was estimated to be ~10^−8^ per nucleotide. While promoters have on average *x* ≈ 3.6 substitutions per nucleotide, for some of them this number can be much higher. Therefore, even with the MAHDS method, there is a need for promoter classification. In studies [24,31], 500 sequences were randomly selected from the promoter database for a given organism. Multiple alignment was performed on these sequences and a position weight matrix (PWM) was calculated. Such a PWM was used as a class matrix. A promoter class consisted of sequences that had a significant alignment with this PWM. Already-classified sequences were removed from the database and the process was repeated until the class size was less than 100 sequences. This resulted in 25 classes combining 17,787 promoter sequences for the *A. thaliana* genome [24] and 5 classes combining 2458 promoter sequences for the *O. sativa* genome [31]. While this method works, it produces a large number of non-optimized classes. Each set of 500 promoters could contain sequences with *x* > 4.4, which would reduce the promoter class specificity of the PWM. Promoter regions from −499 to +100 were used for PWM generation. However, there is a strong triplet periodicity in the +1 to +100 region [32], which may affect the classification of the promoter sequence and thus the accuracy of promoter prediction.

In this study, we describe a new method for promoter sequence classification based on a genetic algorithm and the MAHDS method [24]. It allowed us to divide the human promoters into four classes, containing more than half of the promoters from the EPD database [12]. Then, using dynamic programming in the same way as in [31], we performed a potential promoter sequence (PPS) search in the human genome. A total of 3,065,317 PPSs were found. We analyzed the PPS intersections with known interspersed repeats, genes, introns, and transposable elements from the human genome. It turns out that there is a high probability of PPS intersections with all types of ALU sequences, LTR sequences, and transcript start sites (TSSs).

## 2. Results

### 2.1. Human Genome Promoter Sequence Classes Creation

We shuffled each of the human promoter sequences (29,598 in total). We then created classes from this set of sequences using an algorithm described in Section 4.2. The maximum size of a class was 121 sequences. Since shuffled sequences should not contain common information, we considered such classes as false positives. In order for the promoter classes to contain no more than 20% false positives by volume, we set the minimum promoter class volume to 605 sequences.

Then, we divided 29,598 human genome promoter sequences into classes. We created four promoter classes that met the minimum size requirement. In total, these four classes contained 17,310 promoter sequences, or 58% of the original set. The volume of each class is shown in Table 1.

For each of the created classes, we calculated *R*, a measure of class scalability. *R* is the ratio between the probabilities of finding a promoter sequence in the test sample and in the training sample. The lower *R* is, the more the class is tuned to the specific sequences from the training set. As we can see, the *R* value for the first two classes is close to 1, which means that there is no overfitting. As for the other classes, the higher the class number, the less scalable it becomes. This is probably due to the fact that most of the common features of the promoter sequences were already present in the previous classes. 

Fasta files and PWMs for the four created promoter classes are available as Supplementary Material.

### 2.2. PPS Search in Human Genome

We then searched the human genome for the PPS. We searched each chromosome independently for + and − strands. We also searched inverted and shuffled chromosomes for the PPS. Such sequences have the same nucleotide content as the original chromosomes. The PPS search on randomly shuffled chromosomes was performed to estimate the number of false positives for our search method. The PPS search on inverted chromosomes was essential to estimate the number of symmetric PPSs.

In total, we found 3,065,317 non-intersecting PPSs with *Z* > 6 in the human genome. We also found 76,656 sequences with *Z* > 6 on inverted chromosomes. The number of false positives was 186 for the whole genome. The length of the human genome was approximately 3 × 10^9^ nucleotides. The estimation of the number of false positives was performed for shuffled chromosomes for both + and – strands; thus, the number of false positives for the method used was estimated to be 186/6 × 10^9^ ≈ 3 × 10^−8^ per nucleotide. The number of PPSs for human genome chromosomes, shuffled chromosomes, and inverted chromosomes are shown in Table 2.

**Figure 1:**
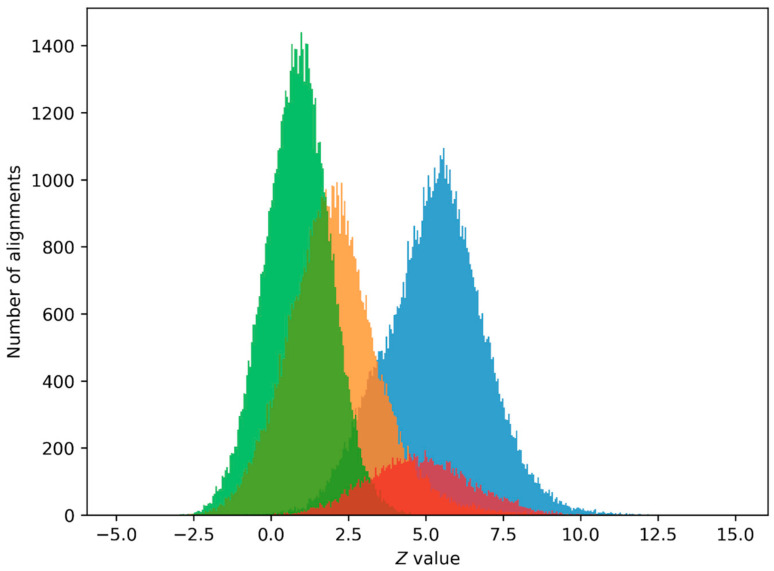
The blue graph is a Z value distribution for alignments of non-intersecting sequences from the 19th chromosome + strand with the first class matrix *W*_1_(*i*, *j*) that are at least 490 nucleotides long. The orange graph is a Z value distribution for alignments of sequences from the inverted 19th chromosome + strand, the green graph is a Z value distribution for alignments of sequences from the shuffled 19th chromosome + strand, and the red graph is a Z value distribution for alignments of sequences from the *Pr* set (Section 4.1), which contains 29,598 human promoter sequences.

**Figure 2:**
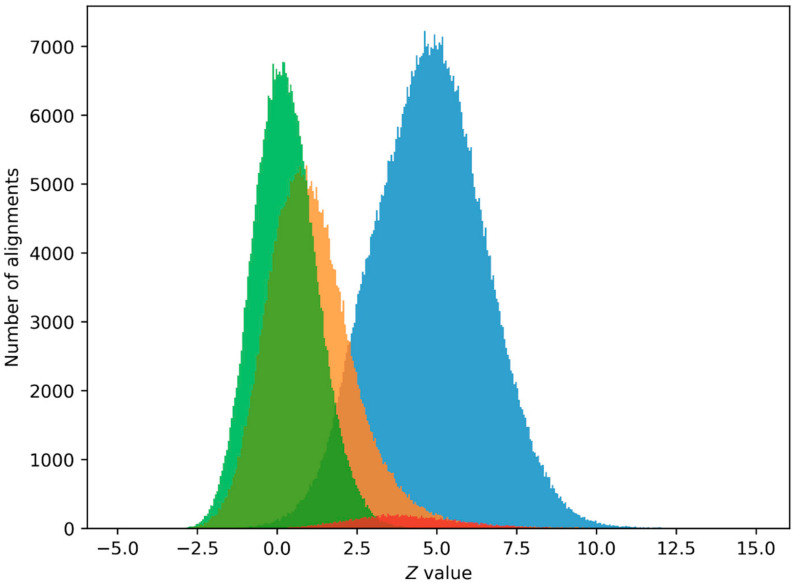
The blue graph is a Z value distribution for alignments of non-intersecting sequences from the 19th chromosome + strand with the second class matrix *W*_2_(*i*, *j*) that are at least 490 nucleotides long. The orange graph is a Z value distribution for alignments of sequences from the inverted 19th chromosome + strand, the green graph is a Z value distribution for alignments of sequences from the shuffled 19th chromosome + strand, and the red graph is a Z value distribution for alignments of sequences from the *Pr* set (Section 4.1), which contains 29,598 human promoter sequences.

We also calculated Z value distributions for alignments of non-intersecting sequences from the 19th chromosome + strand, the shuffled 19th chromosome + strand, and the *Pr* set with first and second class matrices to select the minimum *Z* value for positive PPS prediction. Such distributions are shown in Figure 1 and Figure 2.

We chose the minimum level Z > 6 for positive PPS prediction as the closest integer to the upper limit of the Z value distribution for alignments of sequences from the shuffled 19th chromosome with the class matrices (Figure 1 and Figure 2). It can be seen that the Z value distribution for alignments of sequences from the 19th chromosome and the Z value distribution for alignments of promoter sequences from the *Pr* set are not offset. Z value distributions for other chromosomes and other class matrices are similar to these, with minor changes.

### 2.3. PPS Intersection with Annotated Sequences from the Human Genome

We studied the intersection of human genome PPS coordinates with different types of annotated sequences. For PPS intersections with a *j*-type of annotated sequences, the *Zr*(*j*) coefficient represents the deviation from an expected number of intersections with randomly distributed PPSs in the human genome. Near-zero *Zr*(*j*) values are indicative of a random intersection that does not have statistical significance. Large positive values indicate a positive correlation between PPSs and *j*-type sequence positions. Large negative values indicate a negative correlation between the positions of PPSs and *j*-type sequences.

Coordinates of true promoters and promoters for non-coding genes were obtained from the EPD database [12,13,33]. Annotated exon and intron coordinates for the human genome were obtained from the Ensembl database [34,35]. Repeat and transposable element coordinates were obtained from the Dfam database [36,37]. TSS coordinates were obtained from the refTSS v3.3 database [38,39]. This database contains TSS coordinates from several sources, including NCBI Genes, GENCODE, and ENSEMBL.

The results of the PPS intersection with various annotated sequences are shown in Table 3. 

The intersection of PPSs with the promoter set has a rather large value of *Zr* = 57, indicating its high statistical significance. However, the predicted PPSs only intersect with 10,755 true promoters, while it is possible to predict 17,310 true promoters using four PWM matrices for promoter classes. We assume that the reason is that the sites with higher alignment weights were found in close proximity to some of the true promoters. The presence of such sites may indicate the existence of multiple promoters for a gene [40] or alternative reading frames [41]. As a control, we intersected PPSs with a set of true promoters from the alternative strand. Such an intersection has a negative value of *Zr* = −12, indicating a lack of positive correlation, as it should do.

We also intersected PPSs with a set of promoters for non-coding RNA genes. Such genes can use different RNA polymerases for transcription. This intersection has a rather low positive value of *Zr* = 8, despite the fact that a large proportion of RNA genes use RNA polymerase II for transcription. We assume that this is due to the difference in use of regulatory elements.

The intersection of PPSs with TSSs has a value of *Zr* = 58, which is close to that of true promoters. At the same time, we found intersections with only 50,895 out of 223,949 TSSs from the refTSS database [39]. The reason for this is that the promoter classes we created do not include 42% of the promoters for protein-coding genes. Also, some of the TSSs may be related to RNA polymerase III activity, and for such promoters the search method is not effective because PWMs were created for RNA polymerase II promoters.

Among the PPS intersections with different types of repetitive sequences, the highest *Zr* values were obtained for intersections with Alu elements. This may seem surprising since Alu elements contain the RNA polymerase III promoter [42]. However, we found that more than 75% of the PPSs intersecting with AluS, AluY, or AluJ do not contain their promoter region. Therefore, such high *Zr* values are not due to the promoter structure, but to the structure of the rest of the Alu elements. Alu elements are relatively rich in CpG residues [42], while about 70% of human proximal promoters contain a CpG island [43]. Alu elements have also been found to host a number of transcription factor binding sites [44] and may contain even more. In several cases, Alu elements have been found to influence the expression of nearby genes [44]. At the same time, Alu elements prefer gene-rich regions of the genome [45] and therefore have a high chance of random prediction. Located within genes, Alu elements have been implicated in alternative splicing, translation regulation, and RNA editing [46].

A large value of *Zr* = 53 was also obtained for long terminal repeats (LTRs), which have also been found to be involved in gene expression regulation [47]. All other types of repetitive sequences with high positive *Zr* values are related to either Alu or LTR. 

### 2.4. Comparison with Existing Methods

We compared our method with several existing promoter prediction methods: FPROM [48], TSSW [49], and NNPP [50]. For each method, we generated three sets of sequences. *Set*_1_ contained randomly selected promoter sequences with coordinates −499:20 from the EPD database [33]. *Set*_2_ consisted of random 520 nucleotide sequences with the same nucleotide distribution as the human genome. *Set*_3_ consisted of randomly selected PPSs obtained in this study. Volumes of these sets were denoted as *N_s_*. The results of applying the FPROM, TSSW, and NNPP methods to these sets are shown in Table 4.

Here, positive predictions from *Set*_1_ represent true positives, positive predictions from *Set*_2_ represent false positives, and the number of positive predictions from *Set*_3_ represents the degree of correlation between the studied method and ours.

FPROM is based on a linear discriminant function including functional motifs and oligonucleotide composition description. The method includes two independent functions for TATA and non-TATA promoters. The sensitivity threshold was set at sensitivity = 0.6 for both types of promoters. Here, Ns = 1000 sequences. This method predicts promoters from *Set*_1_ relatively well, as 327 out of 1000 promoter sequences were found. The number of found PPSs from *Set*_3_ was 24, which is significantly more than the 11 false positives found in *Set*_2_.

TSSW recognizes the PolII promoter region and transcription start site. It does not require any parameters. This method gave results comparable to FPROM (Table 4).

NNPP is a method for transcription start site search using a neural network trained on a set of promoters from human and *Drosophila melanogaster* genomes. The minimum promoter score was set to 0.9. NNPP appeared to be incapable of promoter prediction, as the number of true positives and false positives was almost equal (Table 4).

Our method has a higher number of true positives and a lower number of false positives than both FPROM and TSSW, making it more accurate than both. Both FPROM and TSSW make significantly more positive predictions for sequences from *Set*_3_ than for sequences from *Set*_2_. This suggests a positive correlation between these two methods and our method. Sequences predicted by both our method and one of these methods have a much higher chance of being true promoter sequences.

## 3. Discussion

The previous version of the promoter classification algorithm [24] was started by randomly selecting 500 promoters from the promoter set *Ps* for a given organism. An optimal position weight matrix *W*(*i*, *j*) was then computed using the MAHDS method. Pairwise alignments were generated for each sequence from the *Ps* set with the selected position weight matrix *W*(*i*, *j*), and the *Z* value was calculated for each of the pairwise alignments. Promoters whose alignments with the position weight matrix *W*(*i*, *j*) had Z > 5.0 were grouped into a promoter class and *W*(*i*, *j*) became its class matrix. Then, all of the promoters of the created class were removed from the *Ps* set and the procedure was repeated until the size of the created classes became less than 100 sequences. Such an algorithm was used to classify the promoters from the *A. thaliana* and *O. sativa* genomes [24,31].

Due to the random selection of 500 promoters to be used as seed for each class, such sets could contain sequences with *x* > 4.4. This would result in a suboptimal position weight matrix, as the MAHDS method is only able to generate statistically significant multiple alignments for sequences with *x* < 4.4 [24,30], thus reducing the specificity of the PWM. In this study, we replaced the promoter classification algorithm with a new one based on a genetic algorithm. Here, we created *Ko* = 10^2^ seeds for each class. Each seed *Or*(*k*) was considered as an organism. The purpose of using the genetic algorithm was to create a seed with the largest possible *F_m_* value, meaning that it would contain the most similar promoters. To do this, organisms were subjected to crosses, which created a new organism consisting of sequences from two other organisms, and mutations, which replaced several sequences in the organism with sequences from the *Pr* set. The best-performing organism became the seed for the promoter class. In this study, the classes contained promoters with *Z* > 6.0. In this way, the specificity of the *W*_1_(*i*, *j*) matrix, which is a promoter class PWM, was significantly improved.

We created four classes of promoter sequences, covering 17,310 of the 29,598 sequences in the EPD database. For each of the classes, we calculated the scalability measure *R*, which shows the ratio between the probability of finding a promoter sequence in the test set and in the training set. The *R* values for the four classes were between 1.077 and 0.648, and for each class, the *R* value was lower than for the previous one. This meant that the position weight matrices for the four promoter classes combined most of the common features of human promoter sequences.

We compared our method with several existing promoter prediction methods: FPROM [48], TSSW [49], and NNPP [50]. Our method showed both a lower number of false positives and a higher number of true positives than any of the methods studied. However, PPSs that are positively predicted via both our method and one of the other promoter prediction methods have an increased chance of being true promoters.

We searched 24 chromosomes from the human genome and found 3,065,317 PPSs, with an estimated number of false positives at 3 × 10^−8^ per nucleotide, which is several orders of magnitude lower than any other existing promoter search method [51]. We analyzed the PPSs by intersecting their positions with the positions of different types of annotated sequences from the human genome. For each such intersection, we calculated the *Zr* value, which indicated the deviation from an expected number of intersections if the PPSs were randomly distributed in the genome. The intersections of PPSs with true promoters had a value of *Zr =* 5, indicating their high statistical significance. Such an intersection confirms the effectiveness of our promoter prediction method. The intersection with TSSs had a similar value of *Zr =* 58. Those PPSs that intersect with TSSs have a high probability of being true promoters. Thus, an experimental verification of some selected PPSs can be suggested as future work.

The intersection of PPSs with intron sequences had a value of *Zr =* 60, and that with exon sequences was *Zr =* 39. This distribution of PPSs across genes suggests the presence of unknown regulatory pathways related to alternative splicing [52] or alternative reading frames [41]. High *Zr* values for intersections with Alu elements are also evidence for the presence of alternative splicing, referred to as Alu exonization [42]. This phenomenon is widespread, affecting hundreds of human genes. As a result, there may be many more promoter sequences in the human genome than previously thought.

Out of a total of 3,065,317 potential promoter sequences that we found, most of them intersected with known promoters, genes, transposable elements, or dispersed repeats, but 1,241,206 of them were located in non-annotated regions of the human genome. Such PPSs located in non-annotated regions may be associated with unknown copies of known repetitive sequences or transposable elements or unknown genes. They may also indicate the location of microRNAs [53,54]. In this sense, the study of such 1,241,206 PPSs may be of interest for the regulation of the genetic activity of human cells [55].

## 4. Materials and Methods

### 4.1. Promoter Sequences

We used 29,598 human genome promoter sequences from the EPD database [33]. We called this set *Pr* and its elements were denoted as *Pr*(*i*), *i* = 1, 2, …, 29,598. The sequences we used were in the range of −499 to +20, which was chosen to reduce the influence of triplet periodicity [32] that occurs in coding genomic regions on the PWM. We used the GRCh38 release 103 human genome assembly [34].

### 4.2. Classification of Promoter Sequences from the Human Genome 

The task was to include as many promoter sequences from the *Pr* set as possible in a single class. At the same time, there needed to be a minimum number of promoters from other classes in the created class. To solve these tasks and to classify promoter sequences, we developed a mathematical method based on a genetic algorithm.

To speed up the computation, we created a smaller training set *Pr*_1_ containing 10^4^ randomly selected promoter sequences from the *Pr* set. We also created a second set of promoter sequences, *Pr*_2_, containing sequences from the *Pr* set that were not selected in the *Pr*_1_ set. This set would be used as a test set to analyze the reproducibility of the promoter search method. 

After that, we created an “organism” for the genetic algorithm. It had *Po* = 10^2^ randomly selected promoter sequences from *Pr*_1_ set. In this way, we created *Ko* = 10^2^ organisms and named them *Or*(*k*), *k* = 1, 2, …, *Ko*. For each organism *Or*(*k*), multiple alignment $Al_{k}(i,j)$ was performed using the MAHDS method at http://victoria.biengi.ac.ru/mahds/auth. Here, *i* is an index of a promoter in the organism *Or*(*k*)*, i* = 1, 2, …, *Po* and *j* = 1, 2, …, *L*, where *L* is the length of the multiple alignment. Columns with a number of bases less than *Po*/2 were deleted from the multiple alignment $Al_{k}(i,j)$. The result was a transformed multiple alignment $At_{k}(i,j)$ with a length of *L_t_*. *L_t_* ≤ *L*. Then, a frequency matrix *M_k_*(*j,l*) with *j* = 1, …, *L_t_* and *l* = 1, 2, …, 16 was calculated for the multiple alignment $At_{k}(i,j)$.

*M_k_*(*j*, *f*(*At_k_*(*i*,*j*)) + 4(*f*(*At_k_*(*i*,*j* + 1)) − 1)) = *M_k_*(*j*, *f*(*At_k_*(*i*,*j*)) + 4(*f*(*At_k_*(*i*,*j* + 1)) − 1)) + 1 (1)

When filling the *M_k_*(*j,l*) matrix, *i* varies from 1 to *Po* and *j* varies from 1 to *L_t_*. *f*(*a*) = 1, *f*(*t*) = 2, *f*(*c*) = 3, and *f*(*g*) = 4, where *a*, *t*, *c* and *g* are DNA bases. If *At_k_*(*i*, *j*)) or *At_k_*(*i*, *j* + 1) is equal to *, then the *M_k_*(*j,l*) matrix remains unchanged. Next, we computed the PWM matrix—*V_k_*(*i*, *j*)—using the *M_k_*(*i*, *j*) matrix:

(2) $$V_{k}\left(i,j\right)=\frac{M_{k}\left(i,j\right)-Kp(i,j)}{\sqrt{Kp(i,j)(1-p(i,j))}}$$

Here, *i* varies from 1 to *L_t_*, *j* varies from 1 to 16, $p\left(i,j\right)=x\left(i\right)y\left(j\right)/K^{2}$,$x(i)=\sum_{j=1,16}M_{k}(i,j)$, $y(j)=\sum_{i=1,L_{t}}M_{k}(i,j)$, and $K=\sum_{i=1,L_{t}}\sum_{j=1,16}M_{k}(i,j)$. Then, we transformed the *V_k_*(*i*, *j*) matrix so that the *R*^2^ and *K_d_* parameters were the same for all of the matrices with different *k* values. $R^{2}=\sum_{i=1,L_{t}}\sum_{j=1,16}V_{k}^{2}(i,j)$ and $K_{d}=\sum_{i=1,L_{t}}\sum_{j=1,16}V_{k}(i,j)p_{1}(i)p_{2}(j)$. We chose *K_d_* = 0 and *R*^2^ = 45,000. Here, *p*_1_(*i*) is 1/*L_t_* for each *i*;$p_{2}\left(k\right)=p(l)p(m)$, where *p*(*l*) and *p*(*m*) are the probabilities of *l*-type or *m*-type nucleotides in the nucleotide pair (*l,m*∈{*a*,*t*,*c*,*g*}): *p*(*l*) *= q*(*l*)*/L_A_*, where *q*(*l*) is the number of *l*-type nucleotides in *Al_k_*, and *L_A_* is the length of all of the sequences in the multiple alignment *Al_k_*. The matrix transformation procedure is described in detail in [56]. The transformed matrix has *R*^2^ = 45,000 and *K_d_* = 0. After the transformation, we obtained the *W_k_*(*i*, *j*) matrix. Such a matrix transformation is necessary so that the similarity function *F* (Section 4.3) has a similar distribution when different matrices *W_k_*(*i*, *j*) [56] are aligned with nucleotide sequences.

Next, we created local alignments for each of the promoter sequences from the *Pr*_1_ set with the *W_k_*(*i*, *j*) matrix. The alignment algorithm is described in Section 4.3. Then, we estimated the statistical significance of the alignment using the Monte Carlo method and calculated the *Z*(*k*) value. This procedure is described in Section 4.4. We calculated the number of promoter sequences *N*(*k*) from the *Pr*_1_ set with *Z* > *Z*_0_ and an alignment length greater than 490 nucleotides. The number *N*(*k*) is an objective function for an organism *Or*(*k*). The calculation of *N*(*k*) was performed for each *k* from 1 to *Ko*.

Then, we ranked *N*(*k*) in descending order and reordered *Or*(*k*) accordingly. *k* varied from 1 to *Ko*. This meant that *N*(1) would be the largest of all *N*(*k*) and would correspond to an *Or*(1) organism. For the *Or*(1) organism, we also created local alignments of each of the promoter sequences from the *Pr* set with the *W*_1_(*i*, *j*) matrix. For these alignments, the broader promoter region −655:+176 was used to find sequences shifted by up to 30% of their length, relative to the *W*_1_(*i*, *j*) matrix. The number of alignments with *Z* > *Z*_0_ and a length greater than 490 nucleotides was called *N*_f_. The same method was used to determine the number of alignments with a broader promoter region of sequences from the *Pr*_1_ and *Pr*_2_ sets. The obtained numbers of alignments with *Z* > *Z*_0_ and a length greater than 490 nucleotides were designated as *N*_1_ and *N*_2_, respectively.

Next, we removed 10 organisms *Or*(*k*), where *k* was in the interval from *Ko*-9 to *Ko*. These were the 10 organisms with the lowest *N*(*k*). Then, we generated 10 new *Or*(*k*). To generate a pair of new organisms, we selected 2 parent organisms with *k* = *k*_1_ and *k* = *k*_2_, *k*_1_ ≠ *k*_2_. The selection of *k*_1_ and *k*_2_ was performed randomly and the probability of selecting *k*_1_ and *k*_2_ was proportional to the values of *N*(*k*_1_) and *N*(*k*_2_).

Two new organisms (children) *Or* were created due to the two-point crossover of the parent organisms. Two coordinates *i* and *j* were randomly chosen in the interval from 1 to *Po*. The first child contained promoter sequences from 1 to *i* − 1 and from *j* + 1 to *Po* of the organism *Or*(*k*_1_) and promoter sequences from *i* to *j* of organism *Or*(*k*_2_). The second child contained promoter sequences from 1 to *i* − 1 and from *j* + 1 to *Po* of organism *Or*(*k*_2_) and promoter sequences from *i* to *j* of organism *Or*(*k*_1_). Each of the children was subjected to 5 mutations. Each of the mutations could, with a probability of 50%, either swap the order of two promoter sequences in the child or replace one of the promoters with a new one chosen randomly from the *Pr*_1_ set. However, each promoter sequence from the *Pr*_1_ set cannot be present more than once in any organism *Or*, including children. Thus, if a promoter is present more than once in a child, all of its repeats will be replaced by randomly selected sequences from the *Pr*_1_ set.

In total, 5 pairs of parent organisms were selected and 10 children were created. For each of the 10 children, a position weight matrix *W_k_*(*i*, *j*) was created and the *N*(*k*) value was calculated. Then, all organisms were ranked in descending order of *N*(*k*), and for the *Or*(1) organism, the *N*_f_, *N*_1_, and *N*_2_ values were calculated. Then, 10 organisms *Or*(*k*) with *k* in the interval from *Ko*-9 to *Ko* were removed and 10 child organisms were created. This process was repeated until *N*_f_ did not increase for 60 cycles.

After the genetic algorithm finished, the *W*_1_(*i*, *j*) matrix became the promoter sequence class matrix and the *N*_f_ promoter sequences were grouped into one class. The dimension of the matrix was 16 × *L_t_*. The *N*_1_ and *N*_2_ values were used to calculate the scalability of the promoter search with the *W*_1_(*i*, *j*) matrix. The size of the *Pr*_1_ set *S*_1_ was 10^4^ sequences and the size of the *Pr*_2_ set *S*_2_ was equal to the size of the *Pr* set minus 10^4^. The scalability coefficient $R=\frac{N_{1}\;S_{2}}{N_{2}\;S_{1}}$ was a ratio of the probability of finding an alignment with a promoter sequence from the *Pr*_2_ set to the probability of finding an alignment with a promoter sequence from the *Pr*_1_ set.

All sequences contained in the created class were removed from the *Pr* set and the algorithm was repeated for the next class. New classes were created until the last class size *N*_f_ was smaller than *N*_0_. The value of *N*_0_ was determined by classifying random sequences. The exact value is calculated in Section 2.1.

### 4.3. Alignment of Promoter Sequences with Position Weight Matrix W(i, j)

*S*_1_ is a promoter sequence of length *L*_1_. We created the sequence *S*_2_, which contained the numbers 1, 2, …, *L_t_*. *L*_1_ = 520 if the promoter sequence is selected from the *Pr* or *Pr*_1_ sets. If the alignment is made for a wider promoter region from −655 to +176 nucleotides, then *L*_1_ = 832. For the local alignment construction, we calculated the alignment matrix *F*(*i*,*k*) [57]. Here, the *S*_1_ sequence is located along the *X* axis and the *S*_2_ sequence is located along the *Y* axis. To calculate the local alignment, we filled the matrix zero column and zero row with zeros. *F*(0,*k*) = *F*(*i*,0) = 0, *F*_x_(0,*k*) = *F_x_*(*i*,0) = 0, *F*_*y*_(0,*k*) = *F_y_*(*i*,0) = 0 for each *i* from 1 to *L*_1_ and *k* from 0 to *L_t_*. Then, we filled the *F* matrix first column and first row:

(3) $$F(1,k)=max\left\{\begin{array}{l} F(0,k-1)+B(S_{2}(k),S_{1}(1))\\ F_{x}(0,k-1)+B(S_{2}(k),S_{1}(1))\\ F_{y}(0,k-1)+B(S_{2}(k),S_{1}(1))\\ 0 \end{array}\right.$$

(4) $$F(i,1)=max\left\{\begin{array}{l} F(i-1,0)+B(S_{2}(1),S_{1}(i))\\ F_{x}(i-1,0)+B(S_{2}(1),S_{1}(i))\\ F_{y}(i-1,0)+B(S_{2}(1),S_{1}(i))\\ 0 \end{array}\right.$$

(5) $$F_{x}(1,k)=0$$

(6) $$F_{x}(i,1)=0$$

Here, *i* varies from 1 to *L*_1_, and *k* varies from 0 to *L_t_*. Such filling of the first column and the first row is caused by the fact that we need to consider correlations between neighboring nucleotides when creating the alignment. It was impossible to do this for the first row and column, and so they were filled without the *W*(*i*,*j*) matrix and nucleotide correlation. Therefore, we used the *B*(*i*,*k*) matrix instead of the *W*(*i*,*j*) matrix:

(7) $$B(i,k)=0.25\sum_{j=1,4}^{4}W(i,j+(k-1)*4)$$

The other rows of the *F* matrix were filled, as shown below:

(8) $$F(i,k^{})=max\left\{\begin{array}{l} F(i-1,k-1)+W(S_{2}(k),n)\;\;\mathrm{if}\;t=1\\ F_{x}(i-1,k-1)+W(S_{2}(k),n)\;\;\mathrm{if}\;t>1\\ F_{y}(i-1,k-1)+B(S_{2}(k),S_{1}(i))\;\;\mathrm{if}\;t=0\\ 0 \end{array}\right.$$

(9) $$F_{x}(i,k)=max\left\{\begin{array}{c} F(i-1,k)-d\\ F_{x}(i-1,k)-e \end{array}\right.$$

(10) $$F_{x}(i,k)=max\left\{\begin{array}{c} F(i-1,k-1)-d\\ F_{x}(i-1,k-1)-e \end{array}\right.$$

Here, *i* varies from 1 to *L*_1_ and *k* varies from 0 to *L_t_*; *d* = 25; *e* = 6. At the same time, for each (*i*,*k*), we calculated *n* = *S*_1_(*j*) + 4(*S*_1_(*i*) − 1)) and *t*. We considered the pairwise correlation of bases when searching for an alignment in the *W* matrix, and so we introduced the parameter *n*. To calculate *n,* we needed to determine the last base added to the alignment before (*i*,*k*). If the last base from the *S*_1_ sequence (the one already added to the alignment) was *s*(*k* − *t*), then *j* = *k* − t and *n* = *S*_1_(*k* − *t*) + (*S*_1_(*i*) − 1) * 4. If *t* = 1, it corresponded to a diagonal shift in the *F* matrix and there was no deletion in the *S*_1_ sequence. If *t* > 1, it corresponded to a *t* − 1 base deletion in the *S*_1_ sequence. Here, *t* was a parameter that characterized the presence or absence of deletions and their length.

Instead of deletions in the *S*_1_ sequence, there may be deletions in the *S*_2_ sequence. In this case, *t* = 0 and the alignment will skip several columns of the *W*(*i*, *j*) matrix. Since the *W*(*i*, *j*) matrix was created for pairs of nucleotides from adjacent columns, its use is incorrect in the case of deletions in the *S*_2_ sequence. Therefore, for such situations, instead of the *W*(*i*, *j*) matrix, the *B*(*i*, *k*) matrix is used, which lacks correlations between pairs of nucleotides.

After filling the *F*(*i*,*k*) matrix, we found the maximum value of its cells and denoted it as *F_m_* and its coordinates as *i_m_* and *k_m_*. Simultaneously with the *F*(*i*,*k*) matrix, we filled the reverse transition matrix [57]. This allowed us to find the local alignment between the *S*_1_ and *S*_2_ sequences. The start and end coordinates of the local alignment were denoted as *i*_0_, *k*_0_, and *i_m_*, *k_m_*, respectively.

### 4.4. Estimation of the Statistical Significance of a Local Alignment

We estimated the statistical significance of an alignment using the Monte Carlo method. For the Monte Carlo method, we used the *W*(*i*, *j*) matrix, the *S*_1_ and *S*_2_ sequences, the alignment start coordinates *i*_0_ and *k*_0_, the alignment end coordinates *i_m_* and *k_m_*, and the similarity function value *F_m_*. We also created a *Q* set with 13,000 shuffled *S*_1_ sequences. We then created a global alignment of each sequence from the *Q* set with the *S*_2_ sequence. To create the global alignment, we removed 0 from the fourth row in Equations (3), (4) and (8). We also changed the initial conditions for *F*, *F*_x_, and *F*_y_. *F*(0,*k*) = −*kd*; *F*(*i*,0) = −*id*; *F*_x_(0,*k*) = −*kd*; *F_x_*(*i*,0) = −*id*; *F*_*y*_(0,*k*) = −*kd*; and *F_y_*(*i*,0) = −*id* for each *i* from 1 to *L*_1_ and *k* from 0 to *L_t_*. Then, we created a vector *Fr*(*i*) = *F_Q_*_(*i*)_(*i_m_*,*k_m_*) − *F_Q_*_(*i*)_(*i*_0_,*k*_0_). Here, *F_Q_*_(*i*)_ is a similarity function between *S*_1_ and *Q*(*i*) sequences, where *i* varies from 1 to 13,000. Next, the mean $\bar{Fr}$ of *Fr*(*i*) and its variance *D*(*Fr*) were determined. Then, the statistical significance was estimated as $Z=(F_{m}-\bar{Fr})/\sqrt{D(Fr)}$. The use of such a statistical significance estimate *Z* allowed us to minimize the influence of the alignment length. It also allowed us to compare alignments of sequences with different position weight matrices *W_k_*(*i*, *j*). 

### 4.5. Potential Promoter Sequence (PPS) Search in Human Genome

Matrices for classes *W*_1_(*i*, *j*) created in Section 4.2 were used for the PPS search in all of the chromosomes of the human genome. The PPS search was performed separately for each chromosome for + and − strands. When searching for PPSs, we chose a window of 700 nucleotides in length and created local alignments of such window with each of the position weight matrices. In this case, the window was the *S*_1_ sequence with length *L*_1_ = 700. After creating the alignment, we calculated its statistical significance. But, this time, the number of random sequences in the set was 10^3^ and the length of each sequence was 700 nucleotides. Then, the alignment window was moved by 73 nucleotides and all calculations were repeated. We only considered alignments whose length was not less than 490 nucleotides. If the alignment length was less than this, its *Z* = 0.0. Only alignments with *Z* > *Z*_0_ were considered.

A PPS search was performed for each of the created classes. As a result, we obtained vectors *Zp_k_*(*i*) and *Zm_k_*(*i*) for each chromosome, where *i* was the local alignment start coordinate in the chromosome and *k* was the promoter class number. *Zp_k_*(*i*) is a vector for the + chromosome strand, Z*m_k_*(*i*) is a vector for the—chromosome strand. For each vector *Zp_k_*(*i*) and *Zm_k_*(*i*), we found the local maximum coordinate *i*. Here, the local maximum coordinate was *i_m_*, whereby *Zp_k_*(*i_m_*) > *Zp_k_*(*i*) for each *i* from *i_m_* − *L_t_* to *i_m_* + *L_t_*. We denoted the local maximum *i_m_* vectors as *Lp_k_* and *Lm_k_* for the + and − DNA strands, respectively. Priority was given to alignments with the largest *Z* value, regardless of the matrix index.

A PPS search was also performed for inverted chromosome sequences *Cpi* and *Cni* (the sequences were written from the end to the beginning) and for shuffled chromosome sequences *Cpr* and *Cnr*. The PPS search within the *Cpr* and *Cnr* sequences allowed us to estimate the number of false positives for the PPS search within the human genome. The calculation results showed that the number of false positives was about 3 × 10^−8^ per nucleotide.

As a result, we created lists of PPSs for each chromosome. For each PPS found, we show its level of statistical significance *Z*, the coordinates on the chromosome, the DNA strand (+ or −), the position weight matrix *W*_1_(*i*, *j*), and the promoter class represented by this matrix.

### 4.6. Z_0_ Value Selection

We performed a PPS search without a *Z*_0_ threshold on promoter sequences from the *Pr* set and on the shuffled 19th chromosome + strand. We then calculated the number of predictions with different *Z* values and plotted them on a bar graph (Figure 1). Here, predictions from the *Pr* set represent true positives and predictions from the shuffled chromosome represent false positives. The goal in choosing the *Z*_0_ value was to minimize the number of false positives while keeping the number of true positives as high as possible. *Z*_0_ *= 6* was chosen as the minimum integer value, providing a near-zero number of false positives.

### 4.7. Intersection of PPSs with Annotated Sequences from Human Genome

We studied the intersection of the found PPSs with known annotated sequences from the human genome. Such sequences included promoters, exons, introns, transposable elements, tandem and interspersed repeats, and others. We compared the coordinates of the PPSs with the coordinates of the annotated sequences. We considered two sequences to be intersecting if they contained at least 70% of the shorter sequence.

For each annotated sequence type, we also estimated the statistical significance of its intersection with PPSs using the Monte Carlo method. We created 100 sets of random PPS coordinates (*Kor*(*i*), *i* = 1, 2, …, 100) with a distance between them greater than 520 nucleotides. Each set *Kor*(*i*) was intersected with each of the annotated sequence types. *P*r(*i,j*) is the number of intersections between the *j*-type annotated sequences and the sequence set *Kor*(*i*). We then calculated the mean $\bar{Pr(j)}$ and variance *D*(*Pr*(*j*)). The estimate of statistical significance was calculated as $Zr(j)=(Pr(i,j)-\bar{Pr(j)})/\sqrt{D(Pr(j)}$. If *Zr*(*j*) > 6.0, then there was a high probability of a positive correlation between the PPSs and the *j*-type annotated sequence locations. If *Zr*(*j*) < −6.0, then there was a high probability that the PPSs would avoid the type *j* annotated sequences. In other cases, there was only a random intersection between the PPSs and certain annotated sequences.

### 4.8. Comparison with Other Methods for Promoter Prediction

We compared our promoter search method with three different methods for promoter prediction in the human genome. These methods were FPROM, TSSW, and NNPP. Each method was tested on three datasets: *Set*_1_, *Set*_2_, and *Set*_3_. *Set*_1_ contained randomly selected human promoters from the EPD database and the number of positive predictions from *Set*_1_ represents the number of true positives. *Set*_2_ consisted of shuffled promoter sequences. The number of predictions from *Set*_2_ represents the number of false positives. *Set*_3_ contained randomly selected PPSs obtained in this study. The number of positive predictions from *Set*_3_ indicates the degree of agreement with our method.

### 4.9. Computational Resources

All computations were conducted on a system with 4xIntel Xeon Platinum 8270 (104 cores in total). It took about 2 weeks to create the promoter classes and about 4 weeks to scan all 24 chromosomes. 

## 5. Conclusions

We have developed a new method for the classification of promoter sequences based on a genetic algorithm. This method was applied to the classification of promoters of protein-coding genes from the human genome. For each of the four classes created, we calculated PWMs using the MAHDS method [24]. We then searched for potential promoter sequences on 24 chromosomes of the human genome by performing pairwise alignments of chromosome sections with position weight matrices. We found a total of 3,065,317 PPSs. The number of false positives was estimated to be 3 × 10^−8^ per nucleotide. This method was compared with three other methods for predicting promoter sequences within the human genome: FPROM [48], TSSW [49], and NNPP [50]. Our method produced the highest number of true positives and the lowest number of false positives of all the methods studied. The number of false positives for our method is several orders of magnitude lower than for any of the existing methods [51].

## Figures and Tables

**Table 1:** The table shows the volumes *N* of the promoter sequence classes created for the human genome. If the volume of a class is less than 605 sequences, the false positive rate would be higher than 20%. Therefore, we stopped at the 4th class of promoter sequences. *R* is a ratio of the probability of finding an alignment with a promoter sequence from the test sample to the probability of finding an alignment with a promoter sequence from the training sample.

Class	*N*	*R*
1	11,780	1.077
2	3139	1.063
3	1624	0.748
4	767	0.648
5	464	0.447
6	161	0.267

**Table 2:** Number of potential promoter sequences (PPSs) found in different chromosomes of the human genome. + and − are DNA strands.

Chr.№	Number of PPSs		Inverted Chromosome		Random	
	+	−	+	−	+	−
1	133,073	133,437	3374	3252	8	8
2	126,853	128,025	3113	3122	8	9
3	94,729	94,889	2218	2182	3	7
4	81,690	82,344	2125	2049	4	6
5	84,473	84,996	1886	1814	5	7
6	84,193	84,668	2308	2310	6	2
7	82,033	82,165	2242	2152	3	4
8	72,840	72,414	1763	1794	7	5
9	63,751	63,647	1511	1573	2	2
10	73,439	73,275	1739	1724	2	4
11	72,425	72,705	1623	1567	2	4
12	72,578	72,840	1952	2008	9	9
13	45,296	45,786	1133	1131	7	3
14	47,289	47,762	1099	1126	2	3
15	47,703	47,644	1024	987	0	3
16	47,886	47,962	1133	1177	4	1
17	52,660	54,031	1194	1289	3	1
18	39,048	39,345	1031	986	3	5
19	40,174	40,246	1603	1592	1	2
20	39,638	39,855	930	939	2	2
21	20,795	20,958	724	711	0	1
22	25,307	25,335	515	507	0	3
X	69,891	71,404	1987	1837	3	8
Y	10,767	10,767	295	305	2	1
Total	3,065,317		76,656		186	

**Table 3:** Number of PPS intersections with different annotated sequences from the human genome. The calculation of *Zr*(*j*) is described in Section 4.7. Promoters ++/−− mean intersection of PPSs with −499:20 promoter regions for genes from the same strand. Promoters +−/−+ mean intersection of PPSs with −499:20 promoter regions for genes from the opposite strand (e.g., PPSs from + strand with promoters from − strand). NC promoters mean the intersection with the −499:20 promoter region for non-coding genes.

*j*	Annotated Sequences	*Zr*(*j*)	Number of PPSs
1	Promoters ++/−−	57	10,755
2	Promoters +−/−+	−12	4458
3	NC promoters	8	673
4	TSS	58	50,895
5	Exons	39	120,285
6	Introns	60	856,456
7	MIR	−93	66,542
8	AluS	286	196,167
9	AluY	135	38,734
10	AluJ	151	66,919
11	MER	−31	53,258
12	LTR	58	41,789
13	MLT	3	42,846
14	SVA	94	31,567
15	Charlie	−34	6403
16	Tigger	−61	6047
17	MamRep	−16	2885
18	HERV	43	10,963
19	THE	20	12,186
20	L1	−176	111,531
21	L2	−36	70,368
22	L3	−13	4313
23	L4	−22	1313
24	FLAM	21	5604
25	FRAM	12	2526
26	UCON	−12	1502
27	MST	9	7126

**Table 4:** *N_s_* is the number of sequences in *Set*_1_, *Set*_2_, and *Set*_3_. *Set*_1_ contains randomly selected human promoters from the EPD database. *Set*_2_ consists of randomly shuffled promoter sequences. *Set*_3_ contains randomly selected PPSs from this study.

Method	*N_s_*	*Set*_1_	*Set*_2_	*Set*_3_
FPROM	1000	327	11	24
TSSW	400	219	45	75
NNPP	100	47	42	31
Our method	1000	584	0	1000

## Data Availability

Not applicable.

## Supplementary Materials

The supporting information can be downloaded at https://www.mdpi.com/article/10.3390/ijms241612561/s1. The human potential promoter sequence database is available at http://victoria.biengi.ac.ru/cgi-bin/dbPPS/index.cgi. The source code for the project is available at https://github.com/conzaytsev/Potential_promoter_search (accessed on 27 July 2023).
